# Synthesis of Phosphazene-Containing, Bisphenol A-Based Benzoxazines and Properties of Corresponding Polybenzoxazines

**DOI:** 10.3390/polym12061225

**Published:** 2020-05-28

**Authors:** Igor S. Sirotin, Igor A. Sarychev, Viktoria V. Vorobyeva, Anastasia A. Kuzmich, Natalia V. Bornosuz, Denis V. Onuchin, Irina Yu. Gorbunova, Vyacheslav V. Kireev

**Affiliations:** Mendeleev University of Chemical Technology of Russia, 125047, Miusskaya sq. 9, Moscow 125047, Russia; yahoo123-92@mail.ru (I.A.S.); vorobyevavv1995@mail.ru (V.V.V.); akuzmich@muctr.ru (A.A.K.); bornosuz@muctr.ru (N.V.B.); donuchin@muctr.ru (D.V.O.); igorbunova@muctr.ru (I.Y.G.); kireev@muctr.ru (V.V.K.)

**Keywords:** benzoxazine, phosphazene, bisphenol A, hexachlorocyclotriphosphazene, polybenzoxazines

## Abstract

With the aim of obtaining halogen-free polybenzoxazazines with reduced flammability, phosphazene-containing benzoxazines (PhBZ) were synthesized in a two-stage method. In the first stage of the reaction of hexachlorocycotriphosphazene with bisphenol A at molar ratios of 1:12, 1:16, and 1:24, respectively, mixtures of bisphenol and hydroxyaryloxycyclotriphosphazenes were obtained, which mainly contained P_3_N_3_[OC_6_H_4_C(CH_3_)_3_C_6_H_4_OH]_6_. In the second stage, when these mixtures interacted with aniline and an excess of paraformaldehyde in toluene at 80–90 °C, PhBZ containing 20–50% of the phosphazene component with M_w_ 1200–5800 were formed. According to ^1^H and ^13^C NMR spectroscopy, PhBZ contain a small amount of oligomeric compounds with Mannich aminomethylene bridges. With an increase of the content of the phosphazene component, the curing temperature of PhBZ decreases from 242 °C to 215 °C. Cured PhBZ samples with a phosphorus content of more than 1.5% have increased flammability resistance according to UL-94.

## 1. Introduction

Polybenzoxazines (PBZ) are a relatively new class of polymers obtained by thermal ring-opening polymerization of monomeric and oligomeric compounds with oxazine rings without the release of volatile byproducts, which is the main advantage of this class of compounds [1]. Due to the formation of hydrogen bonds in the polymer [2], PBZs have good physicomechanical properties, low water absorption [3], and practically zero curing shrinkage [4]. Despite the reduced combustibility, basic polybenzoxazines (synthesized from A/F bisphenols and aniline) have a burning resistance rating of only V-1 in accordance with UL-94; flame retardancy is required to achieve a V-0 rating.

Over the past 20 years, great success has been achieved in the field of the molecular design of benzoxazines; in particular, functional groups such as allyl [5], maleimide [6], carboxyl [7], propargyl [8], and cyanate ester [9], nitrile [10] and others have been introduced into the structure of these monomers.

One of the methods for the modification of PBZ is the introduction into the structure of the initial monomers of organoelement polyfunctional fragments [11], which increase thermal stability and fire resistance. For example, the introduction of polyhedral oligosiloxanes into the structure of PBZs, along with an increase in thermal stability, contributes to the improvement of the their dielectric properties [12].

Phosphazenes also belong to the class of organo-inorganic compounds [13], the presence of which in polymers lowers combustibility [14] and increases heat resistance and degradation temperature [15]. Phosphazenes have advantages not only in comparison with unsafe halogen-containing flame retardants, but also in comparison with conventional phosphorus-based flame retardants [16,17] Functional phosphazenes contribute not only to reduced flammability, but may also be used to adjust the mechanical properties of the resulting thermosets [18,19], which make them one of the most promising halogen-free flame retardants. Phosphazene-containing benzoxazines synthesized in [20,21,22,23] form high fire resistance compositions during polymerization due to the synergistic effect of phosphorus and nitrogen atoms. However, the typical synthesis methods [20,23,24] of such compounds are multistage (4–5 stages) and complex (as they include the preparation of Schiff base and its subsequent reduction). This reduces the prospects for the use of phosphazene-benzoxazines in applied areas.

In this work, we synthesized oligomeric phosphazene-containing benzoxazines based on bisphenol A with a different content of the phosphazene component by reacting a mixture of oligomeric hydroxyaryloxycyclotriphosphazenes and bisphenol A with aniline and paraformaldehyde. Compared with other known methods for the preparation of phosphazene-containing benzoxazines, the method proposed in this work is more convenient and scalable.

The structure of the precursors and the obtained oligomeric phosphazene-containing benzoxazines was confirmed by ^1^H, ^13^C, and ^31^P NMR spectroscopy, mass spectrometry, and gel permeation chromatography. The thermal characteristics were investigated using differential scanning calorimetry and thermogravimetric analysis.

## 2. Materials and Methods

### 2.1. Starting Materials

Hexachlorocyclotriphosphazene is a white crystalline substance with a melting point (m.p.) of 113 °C [25]. Bisphenol A (PJSC Kazanorgsintez, Kazan, Russia) was purified via repeated recrystallization from chlorobenzene to yield a product with a m.p. of 156.5 °C. Aniline (Sigma-Aldrich, St. Louis, MI, USA), a colorless liquid, was distilled twice under vacuum before use; its boiling point (b.p.) is 184 °C. Paraformaldehyde (Ercros, Barcelona, Spain), a white crystalline solid in the form of prills with a paraformaldehyde content of 91%, was used as received; it has a m.p. 120 °C (decomposes) and a molecular weight of 300–3000. Potassium carbonate (Sigma-Aldrich, St. Louis, MI, USA), a white crystalline substance in the form of powder, soluble in water, was dried in a vacuum at 100 °C before use. Solvents were purified according to known methods, and their physical characteristics corresponded to literature data [26].

### 2.2. Synthesis Methods

#### 2.2.1. Synthesis of Model Monomer BA-a

A 50 mL round bottom flask equipped with a magnetic stirrer and reflux condenser was charged with 10 g (0.0438 mol) of bisphenol A, 8.159 g (0.0876 mol) of aniline, and 16 mL of toluene. The solution was heated to 60 °C until bisphenol A was completely dissolved. Then, 6.071 g (0.184 mol) of paraformaldehyde was loaded. The reaction was carried out at a temperature of 80 °C for 6 h; then, the reaction mass was dissolved in 50 mL of toluene, the lower aqueous layer was separated on a separatory funnel, and the upper toluene solution of the product was washed twice with distilled water. The solution of oligomers in toluene was dried with calcined magnesium sulfate and then filtered, the toluene was distilled off under vacuum, and the product was dried at 100 °C. The yield of light-yellow powdery substance was 97%.

#### 2.2.2. Synthesis of Hydrohyaryloxyphosphazenes (HAP)

Three different HAPs with different HCP: bisphenol A ratios were synthesized. The amounts of reagents used in each case are shown in Table 1.

First, 4 g (0.0115 mol) of hexachlorocyclotriphosphazene, a calculated amount of bisphenol A (Table 1), and 200 mL of acetonitrile were charged into a 500 mL three-neck round-bottom flask. After dissolving the solid reagents, 19.06 g of K_2_CO_3_ (0.138 mol) was added to the solution. The synthesis was carried out at the boiling point of acetonitrile and under stirring for 12 h, sparging the reaction mixture with argon. Next, acetonitrile was distilled off in vacuo, the residue was dissolved in diethyl ether, and a 1 N aqueous HCl solution was poured into the solution until an acid reaction with indicator paper occurred. After the formation of two layers, the upper organic layer was separated on a separatory funnel and washed with water until neutral. It was then dried with calcined magnesium sulfate and filtered; diethyl ether was distilled off under vacuum and the product was dried to constant weight in vacuum. A mixture was obtained consisting of oligomeric hydroxyaryloxphosphazenes and unreacted bisphenol A. The yield of white powder products was 90% of the theoretical maximum.

#### 2.2.3. Synthesis of Phosphazene-Containing Benzoxazines Based on Mixtures of Hydroxyaryloxycyclotriphosphazenes and Bisphenol A

In a 100 mL round-bottom flask were charged 4 g of a HArPh mixture, aniline, the calculated amount shown in Table 1 and 30 mL of toluene. The mixture of oligohydroxyaryloxyphosphazenes and bisphenol A was dissolved in toluene and aniline at a temperature of 110 °C; then, the temperature was lowered to 80 °C and the calculated amount of paraformaldehyde was added (Table 1). The synthesis was carried out at 85–90 °C for 8 h. The resulting solution was washed twice in a separatory funnel with distilled water. The toluene solution of the product was dried with calcined magnesium sulfate, and then filtered, and the toluene was distilled off under vacuum. After drying at 100 °C, the yield of a light-yellow powdery product was 88–90% of the calculated maximum.

The characteristics of the obtained oligomers BP-1, BP-2 and BP-3 are presented hereinafter.

### 2.3. Curing of Benzoxazines

All polybenzoxazines in this work were cured according the following mode: 2 h at 150 °C, 4 h at 180 °C, 4 h at 190 °C, 2 h at 200 °C. Before curing, the samples were degassed at a temperature of 130 °C for 1 h. The completeness of the curing process was monitored by the absence of an exothermic effect on the DSC thermogram.

### 2.4. Methods of Analysis

The and ^1^H, ^13^C and ^31^P NMR spectra were measured with a Bruker AV-600 spectrometer (Bruker Corporation, Bremen, Germany) operating at 600, 151 and 243 MHz respectively. For the NMR experiments, the following parameters were as follows: (^1^H) acquisition time—2.7 s, relaxation delay—1.0 s, number of scans—1, (^13^C) acquisition time—0.45 s, relaxation delay—0.1 s, number of scans—150, (^31^P) acquisition time—0.485 s, relaxation delay—0.5 s, and number of scans—18. The temperature of all measurements was 298 K. The chemical shifts of the signals were calculated relative to the signals of tetramethylsilane (^1^H, ^13^C spectra) and phosphoric acid (^31^P spectra), which were used as internal references. Residual ^1^H resonance from deuterated solvent was used to reference the ^1^H spectra. The ^13^C spectra were referenced through the solvent ^13^C resonance. The ^31^P spectra were referenced through the deuterated solvent lock. Deuterated chloroform (CDCl_3_) was used as a solvent for all benzoxazines and deuterated dimethyl sulfoxide (d-DMSO) was used as a solvent for the hydroxyaryloxyphosphazene samples. The spectra were processed using the MestReNova Lab software package (Version 14.1, MESTRELAB RESEARCH, S.L, Santiago de Compostela, Spain).

Gel-permeation chromatography (GPC) was carried out on a Shimadzu LC-20 Prominence (Kyoto, Japan) chromatograph equipped with refractometric and UV detector (a wavelength of 264 nm,) and a PSS column (SDV; 300 mm × 8 mm; 1000 A, separation within 100–60,000 Da). Tetrahydrofuran (THF) was used as an eluent (1 mL/min). Molecular mass values were estimated with the use of a polystyrene calibration curve.

Spectrophotometric determination of phosphorus was carried out on a Cary-100 instrument (Agilent Technologies, Santa Clara, California, United States). The method for determining phosphorus [27] consists of the preliminary destruction of organic matter with the transfer of phosphorus to the solution in the form of a phosphate ion and its subsequent spectrophotometric determination in the form of a blue phosphorus-molybdenum complex. Method error is ±0.30% abs.

Differential scanning calorimetry (DSC) was performed on a Netzsch DSC 204 F1 Phoenix instrument (Netzsch, Selb, Germany) in a nitrogen atmosphere (20 mL/min) at a heating rate of 10 deg/min on samples weighing ~ 10 mg.

Thermogravimetric analysis (TGA) was carried out on a Netzsch STA Jupiter instrument (Netzsch, Selb, Germany) in corundum crucibles without a cover in air and in an argon stream at a rate of 70 mL/min in a temperature range of 50–900 °C with a heating rate of 20 deg/min. The mass of the samples was 10–16 mg.

A MALDI-TOF mass spectrometric analysis was carried out on the Bruker Auto Flex II instrument (Bruker Corporation, Bremen, Germany).

Liquid chromatography-mass spectrometry (LC-MS) with electrospray ionization (ESI^+^) was performed on an Agilent 1100 instrument (Agilent Technologies, Santa Clara, California, United States) using gradient elution in an acetonitrile-water system on a 4.6 mm Reprosil-Pur Basic C18 250 column with an ion trap LC-MSD-Trap-SL.

Limiting oxygen index (LOI) was determined according ASTM D2863. The sample dimensions were 80 × 10 × 4 mm.

Flammability tests were performed according UL-94 standard. The samples dimensions were 127 × 12.7 × 2 mm.

Tensile strength was determined using a Z010 testing machine (ZwickRoell, Kennesaw, GA, USA).

## 3. Results and Discussion

### 3.1. Synthesis of Model Benzoxazines

It is known that during the synthesis of benzoxazines, side reactions are possible, including the formation of oligomers with Mannich bridges and free phenolic groups. [28]. The synthesis methods that involve the stage of purification from such compounds by washing with an alkaline solution [29,30] are undesirable in our case due to the possible loss of phosphazene oligomers. Therefore, it was necessary to develop a synthesis method that would ensure a high yield of oxazine compounds without a purification step.

**Scheme 1:**
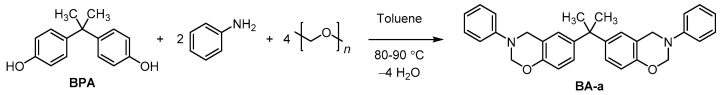
Synthesis of benzoxazine monomer (BA-a) by the reaction of bisphenol A, aniline and paraformaldehyde.

Toluene was chosen as a solvent, as it is one of the most suitable for the solution synthesis of benzoxazines [31,32]. The use of paraformaldehyde seems to be more convenient due to the possibility of its long-term storage and lower reactivity with respect to the primary amine due to the heterogeneity of the reaction, which reduces the likelihood of the formation of oligomers with Mannich bridges. As reported by Liu [33], the effect of various parameters on the synthesis of benzoxazines was studied, and it was found that an increase in temperature above 100 °C leads to the loss of formaldehyde from thermally destructible paraformaldehyde, which does not have enough time to react. Therefore, we synthesized benzoxazine oligomers at 80–90 °C according to the (Scheme 1). On the other hand, during the synthesis of benzoxazines in toluene, water is released, in which some part of formaldehyde also dissolves and does not react, leaving the reaction zone. Therefore, we used a 5% excess of paraformaldehyde relative to the stoichiometric amount, and we also took into account the 91% purity of paraformaldehyde.

With this approach, it is possible to obtain benzoxazine oligomers with a minimum amount of free hydroxyl groups that can be formed by the Mannich reaction, and to avoid washing the reaction products with an alkali solution to remove such oligomers. This step is undesirable, since when washing with alkaline water, a toluene solution containing high molecular weight phenolic oligomers, it is difficult to achieve phase separation.

**Figure 1:**
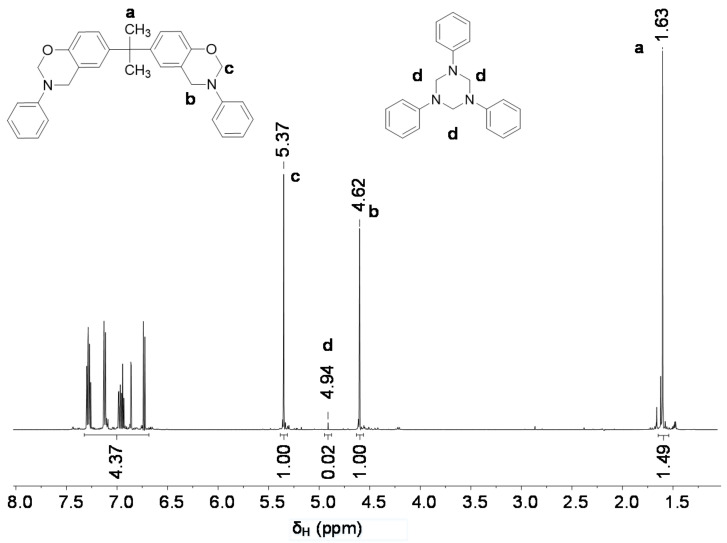
^1^H NMR spectrum of BA-a benzoxazine synthesized according to Scheme 1.

**Figure 2:**
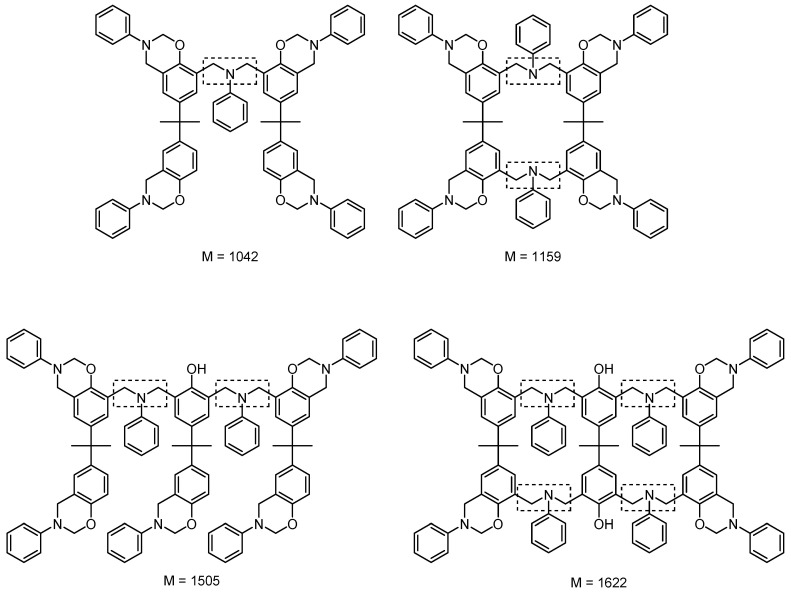
Formulas of dimers and trimers with aminomethylene Mannich bridges (marked with dashed line), the formation of which is possible during the synthesis of BA-a benzoxazine monomers according to Scheme 1.

On the ^1^H NMR spectrum of the reaction products (Figure 1), signals with chemical shifts δ_H_ = 4.62 ppm (s, Ar-C*H_2_*-N) and 5.37 ppm (s, O-C*H_2_*-N) belonged to the oxazine rings; no significant amounts of Ar-C*H_2_*-NPh-C*H_2_*-Ar_—_fragments formed by the Mannich reaction (Figure 2, marked with dashed line), and chemical shifts in the region of δ_H_ = 4.20–4.50 ppm were detected.

Compounds obtained in this way usually contain dimers, trimers and higher molecular weight oligomers [34], the proposed formulas of which are shown in Figure 2.

**Figure 3:**
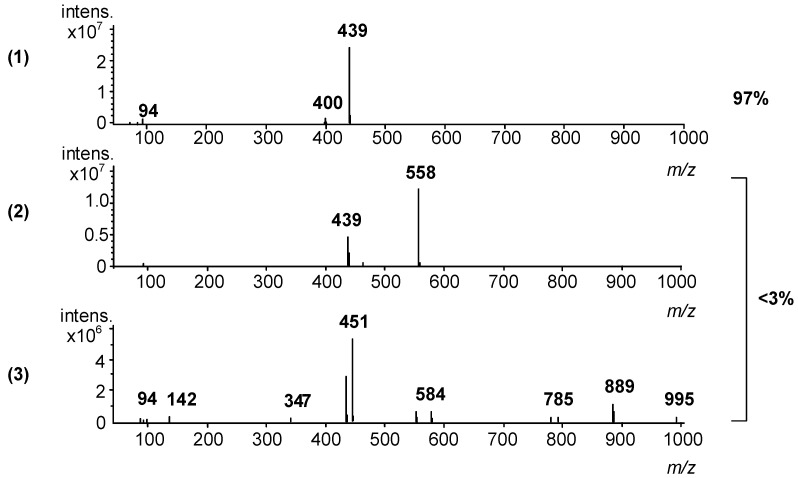
ESI^+^ mass spectra of fractions of benzoxazine synthesized according to Scheme 1. Retention time of fractions: 13.4 (**1**), 15.4 (**2**) and 17.6 min (**3**).

**Figure 4:**
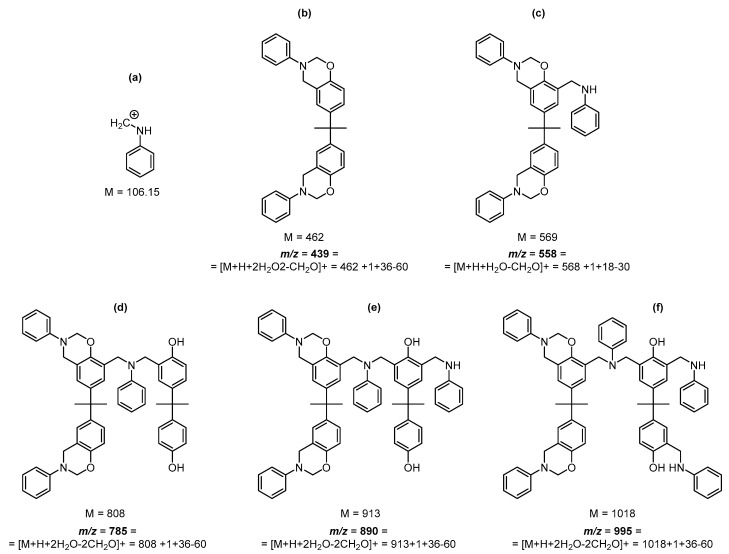
Ion structures and their *m*/*z* values formed during mass spectrometry with electrospray ionization of BA-a benzoxazines obtained according to the Scheme 1.: iminium ion derived from phenylaminomethylene fragment (**a**); fragments with one (**b**,**c**) and two (**d–f**) bisphenol moieties.

Unfortunately, MALDI-TOF mass spectrometry was not suitable for identifying compounds with oxazine rings due to intense destruction under desorption-ionization conditions. Therefore, for the detection of high molecular weight impurities, we used liquid chromatography-mass spectrometry (LC-MS) with electrospray ionization (ESI), which had previously been successfully used for the analysis of 1,3-benzoxazines [35]. The mass spectra of the fractions with the exit time from the chromatographic column 13.4, 15.4, and 17.6 min are shown in Figure 3. The *m*/*z* values of benzoxazines were found using the formulas [M + H + H_2_O − CH_2_O]^+^ or [M + H + 2H_2_O − CH_2_O]^+^, where M is the molecular weight of the fragment depicted in Figure 4a.

Molecular ions with *m*/*z* = 439 (retention time = 13.4 min) correspond to dibenzoxazine according to the formula [M + H + 2H_2_O − 2CH_2_O] (Figure 4b). With a retention time of 15.4 min, a peak with *m*/*z* = 558 appears, which may refer to a molecular ion with two oxazine rings and one aminomethylene bridge [M + H + H_2_O − CH_2_O] + (569 + 1 + 18–30, Figure 4c); the same peak may refer to a fragment from a benzoxazine dimer, due to destruction under the conditions of analysis. The relative intensity of the main component peak (fraction 1) with a retention time of 13.4 min is 97%, which indicates a high purity of the benzoxazine monomer. Other possible ion structures generated as a result of the electrospray ionization and their *m*/*z* values are presented in Figure 4g–f.

Molecular ions with *m*/*z* = 785, 889 and 995 observed in the mass-spectrum of the fraction with a retention time of 17.6 min differ by 104–106 amu, which is close to the mass of the phenylaminomethylene fragment, which is probably formed upon fragmentation of trimeric or higher molecular weight oligomeric benzoxazines.

Thus, when using excess paraformaldehyde in a one-step synthesis, it is possible to obtain benzoxazine monomers with a minimum amount of oligomers with phenolic hydroxyl groups, which can be formed by the Mannich reaction.

### 3.2. Synthesis of Hydroxyaryloxyphosphazene Precursors

It was previously shown that a convenient method for the synthesis of hydroxyaryloxycyclotriphosphazenes is the use of excess diphenol at the stage of the substitution of chlorine atoms in HCP, which is not subsequently removed, but the obtained mixture of hydroxyaryloxycyclotriphosphazenes and diphenol is used for further transformations [36].

**Scheme 2:**
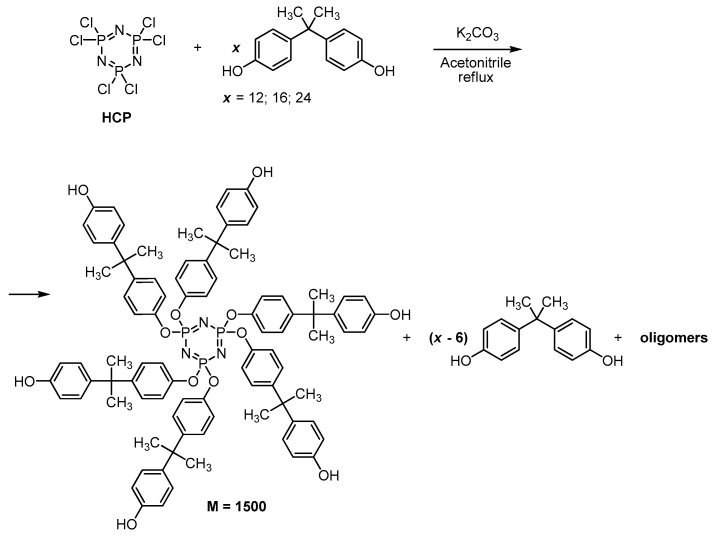
Synthesis of hydroxyaryloxycyclotriphosphazenes (HArPh) by the reaction of hexachlorocyclotriphosphazene (HCP) and bisphenol A with different molar ratio (x = 12; 16; 24).

To obtain phosphazene-containing benzoxazine oligomers, we used hydroxyaryloxycyclotriphosphazenes synthesized by the interaction of HCP with an excess of bisphenol A in the presence of an excess of potassium carbonate in an acetonitrile medium (Scheme 2).

**Figure 5:**
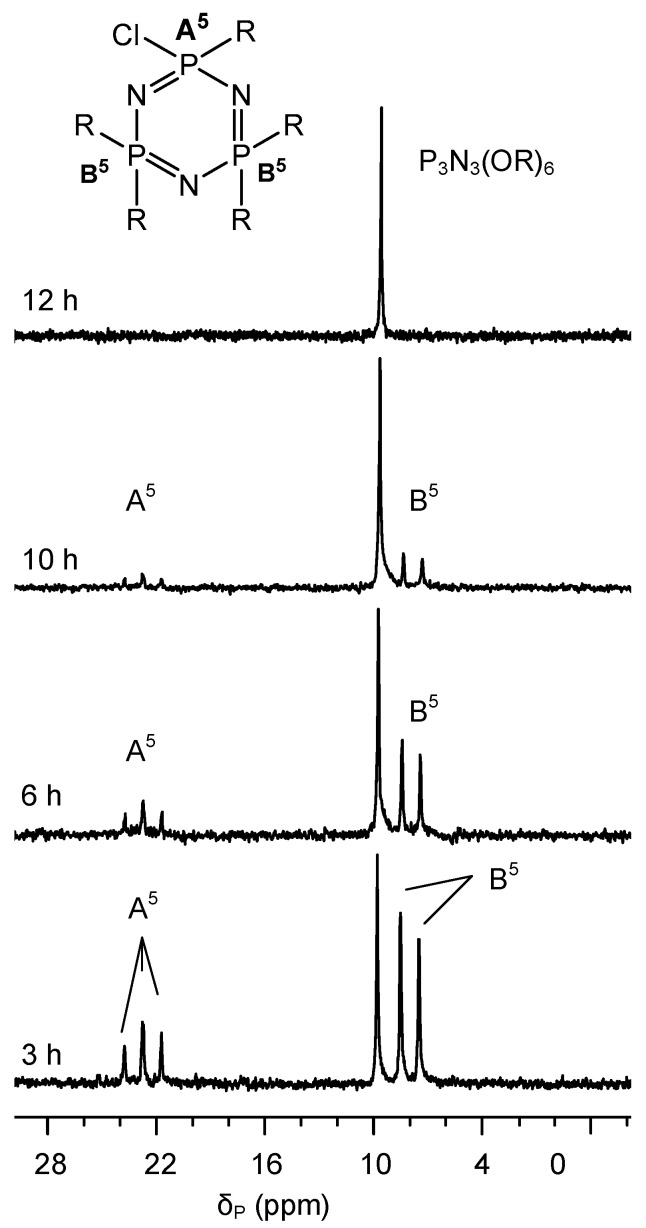
Changes in the ^31^P NMR spectra of the reaction products of HCP and bisphenol A (1:24) with a process duration of 3–12 h.

Three hours after the start of the reaction, the signals of phosphorus atoms in the completely substituted cyclotriphosphazene nucleus at δ_p_ = 9.86 (s) ppm and the signals of the AB_2_ system appeared on the ^31^P NMR spectrum of reaction products (Figure 5) with δ_A_ = 22.96 (t) ppm and δ_B_ = 8.13 (d) ppm, with the latter belong to the pentasubstituted cyclotriphosphazene core. The complete replacement of phosphorus atoms by bisphenol A was achieved after 12 h at all selected molar ratios of HCP: bisphenol A (x = 12, 16, 24).

**Figure 6:**
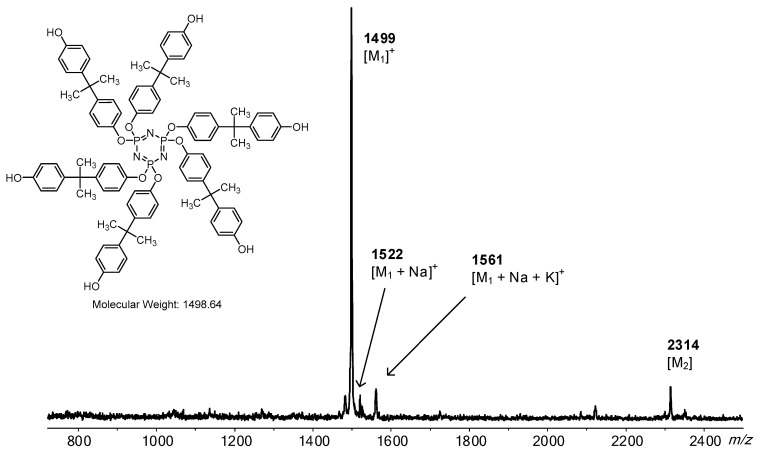
MALDI-TOF mass spectrum of hydroxyaryloxyphosphosphazenes synthesized at a molar ratio of HCP: bisphenol A = 1:12.

On the MALDI-TOF mass spectra of the reaction products obtained with HCP: bisphenol A ratio = 1:12 (Figure 6), two main substances are fixed. The main peak of molecular ion [M_1_]^+^ with *m*/*z* = 1499 refers to the hexasubstituted derivative. This substance is also probably represented by two cationized particles with *m*/*z* 1522 and 1561. The peak of molecular ion [M_2_]^+^ with *m*/*z* = 2314 most probably corresponds to cyclolinear oligomers with three bisphenol bridges between two trimeric cycles, respectively:


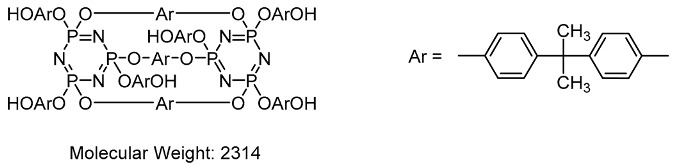


Judging by the intensities of the indicated peak in the MALDI-TOF mass spectra, the number of cyclolinear oligomeric hexahydroxyaryloxycyclotriphosphazenes does not exceed 8%, even when the ratio of HCP: bisphenol A is 1:12.

### 3.3. Synthesis of Phosphazene-Containing Benzoxazines

**Scheme 3:**
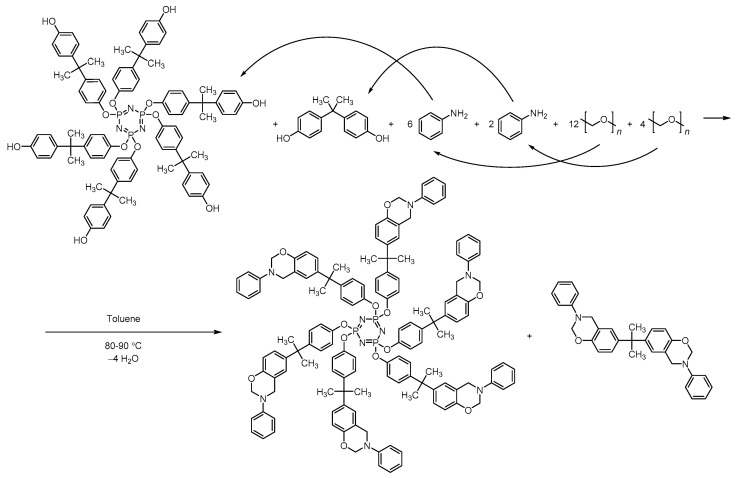
Synthesis of phosphazene-containing benzoxazines by the reaction of mixture HAP with bisphenol A, aniline and paraformaldehyde with different molar ratio HCP: Bisphenol A of 1:24 (BP-1), 1:16 (BP-2) and 1:12 (BP-3).

To obtain phosphazene-containing benzoxazines, we previously used synthesized mixtures of oligomeric hydroxyaryloxycyclotriphosphazenes with bisphenol A with different contents of the phosphazene component at ratios HCP: Bisphenol A of 1:24 (**BP-1**), 1:16 (**BP-2**) and 1:12 (**BP-3**) as the initial reagents (Scheme 3, possible side oligomeric products are not shown). The reaction conditions were similar to those for the previously described synthesis of benzoxazine (BA-a) based only on bisphenol A.

On all ^31^P NMR spectra of phosphazene-containing oligomeric benzoxazines, a singlet signal δ_P_ = 9.83 ppm was observed, indicating that the phosphazene cycle structure remains unchanged. The results of ^1^H and ^13^C NMR spectroscopy are shown in Table 2, which shows that along with the methylene protons signals of the oxazine ring, δ_H_ = 4.62 (s, Ar-CH_2_-NPh-) and δ_H_ = 5.37 ppm (s, O-CH_2_-NPh), and signals of carbon atoms at δ_C_ = 50.70 (s) and 79.13 (s) ppm, respectively, there are low-intensity proton signals δ_H_ = 4.47 (s) ppm and carbon δ_C_ = 41.81 (s) ppm, which indicate the formation of an insignificant amount of oligomers with the Mannich aminomethylene bridge.

**Figure 7:**
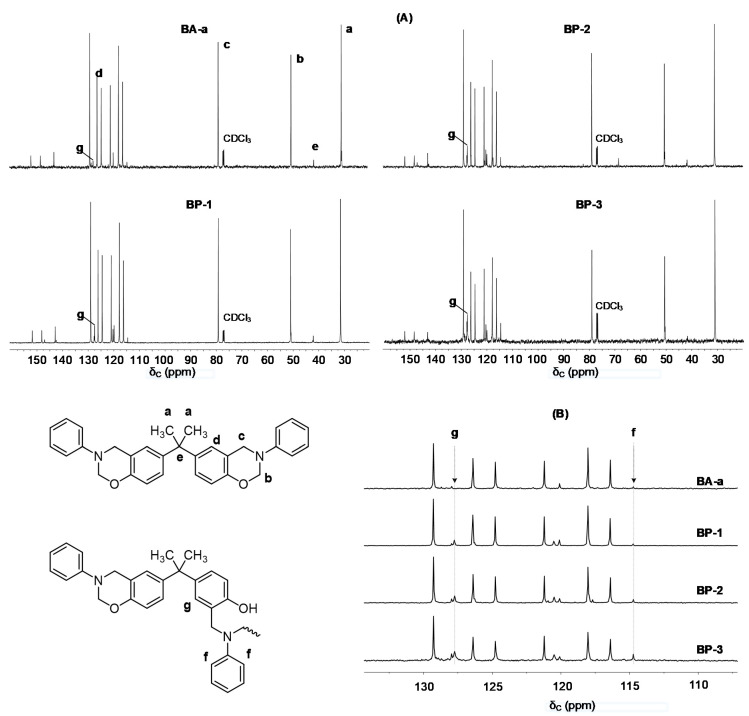
^13^C NMR spectra of benzoxazine oligomers (**A**) and their enlarged fragments (**B**).

On the ^13^C NMR spectra (Figure 7), no differences are found in the signals of the carbon atoms of the Mannich aminomethylene bridge; however, differences arise in the chemical shifts of the carbon atoms in the benzene ring of the bisphenol A (Figure 7). In the case when an oxazine ring is formed, the carbon atom in the *meta* position has a chemical shift of δ_C_ = 126.4 ppm. (“D”), and during oligomerization, it shifts by δ_C_ = 127.7 ppm. This behavior is consistent with that described in the literature for the example of monofunctional benzoxazine monomers [28,37].

As in the case with BA-a, MALDI-TOF mass spectrometry was not suitable for identifying phosphazene compounds with oxazine rings, due to intense destruction under desorption–ionization conditions. We also tried using LC-MS with electrospray ionization to study the high molecular weight phosphazene-benzoxazine fraction in the synthesized products, where 1,3-benzoxazines were also found to undergo fragmentation, but to a much lesser extent when compared to MALDI-TOF. On the ESI^+^ mass spectra (Supplementary information, Figure S5) of the phosphazene-containing benzoxazine BP-1, molecular ions appear with *m*/*z* > 1000 (1116, 1140, 1202, 1289, 1535), which, due to the variety of possible oligomeric structures formed by the phosphazene-containing benzoxazines and the fragmentation of molecules with a large number of oxazine rings, are difficult to decipher; however, if we compare with the ESI^+^ mass spectra of benzoxazine oligomers without a phosphazene component, it can be assumed that molecular ions with *m*/*z* > 1000 belong to phosphazene-containing benzoxazine oligomers.

According to the results of an elemental analysis of all phosphazene-containing products (Table 3), an increased phosphorus content was determined compared to that calculated on the hexasubstituted phosphazene-benzoxazine monomer with six oxazine rings and the bifunctional BA-a monomer, especially in the BP-3 sample. The increased phosphorus content may be due to the presence, in addition to the individual hexazubstituted hydroxyaryloxycyclotriphosphazene based on bisphenol A, of cyclolinear oligomeric hydroxyaryloxycyclotriphosphazenes. The presence of the latter reduces the overall functionality of the phosphazene component at the stage of benzoxazine synthesis, and oligomerization in the phosphazene component at the same stage is possible.

**Figure 8:**
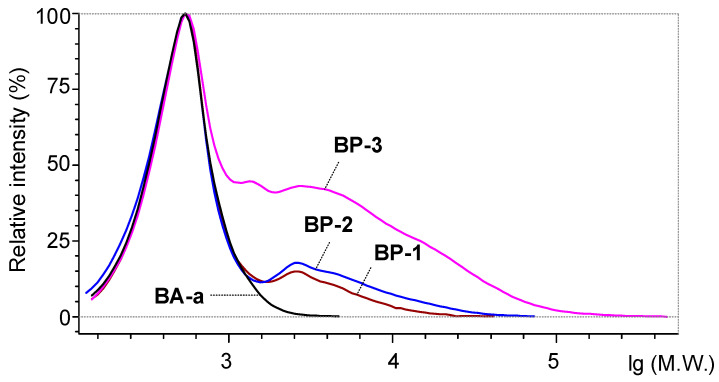
Molecular weight distribution and composition of BA-a benzoxazine and phosphazene-containing benzoxazines according to GPC.

According to gel permeation chromatography (Figure 8, Table 3), the BA-a monomer has M_n_ = 480, M_w_ = 610. The somewhat overestimated values according to GPC in comparison with the results of mass spectrometry are probably due to the limited applicability of standard polystyrene calibration. Nevertheless, the obtained values of the molecular masses of BA-a are in satisfactory agreement with the actual values of 462, which allows one to estimate with sufficient accuracy the molecular weight characteristics of phosphazene-containing benzoxazines. In samples BP-1 and BP-2, the values of molecular weights and polydispersity index are in good agreement with those calculated under the assumption that the phosphazene component contains predominantly hexafunctional benzoxazine-phosphazene. The molecular weight distribution of high molecular fractions in both samples is practically the same, only their amounts differs. The average molecular weight of the BP-3 sample, as well as the polydispersity index, is significantly higher than in BP-1 and BP-2, which indicates an increased content of cyclolinear oligomers containing two or more phosphazene cycles in the BP-3 sample.

The contents of the phosphazene component in the obtained mixtures (Table 3), determined by gel permeation chromatography and by elemental analysis of phosphorus, are in satisfactory agreement with each other and with the calculated values.

### 3.4. Polymerization of Phosphazene-Containing Benzoxazines and Properties of the Obtained Polybenzoxazines

**Figure 9:**
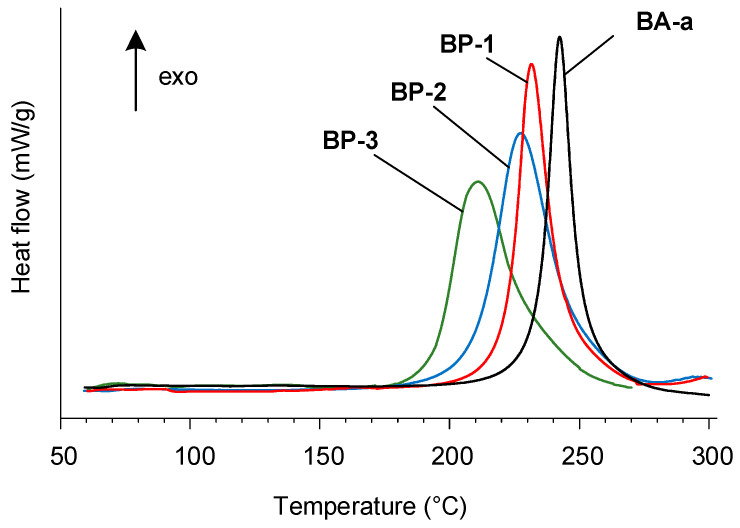
DSC curves of curing of benzoxazine BA-a and phosphazene-containing benzoxazines.

The curing process of a series of obtained phosphazene-containing benzoxazine oligomers and BA-a monomer were studied using differential scanning calorimetry. DSC curves are shown in Figure 9, and the results of their analysis are shown in Table 4.

Table 4 shows that with an increase in the content of the phosphazene component in benzoxazines, the curing start temperature, peak temperature, and the thermal effect of the reaction decrease, which is most likely associated with a decrease in the mass fraction of oxazine rings in the product. As in the case of phosphazene-containing epoxies [18,38], a decrease in the curing temperature is observed, which can be attributed to the catalytic effect of phosphazene fragments, which accelerate the opening of benzoxazine rings. Alternatively, phenolic oligomeric impurities can also contribute to lowering the polymerization temperature.

The glass transition temperature of PBZ (Table 5) was determined on samples preliminarily cured by temperature modes of 2 h at 150 °C, 4 h at 180 °C, 4 h at 190 °C and 2 h at 200 °C, as well as when rescanning a cured sample directly in the DSC device, cooled to normal temperature. The glass transition temperature of the obtained polybenzoxazines did not change significantly with the increase of phosphazene content, but we observed significant differences in the values of T_g_ of the samples cured according to the given mode and obtained by repeated scanning in a DSC instrument. It was revealed that for the determination of T_g_ of PBZ by the DSC method, it is preferable to use previously cured samples. Although when heated at a speed of 10 degrees per minute up to 300 °C, curing occurs completely (which is evidenced by the absence of exothermic effects during repeated scanning), it seems that a defective polymer network is formed, characterized by a lower glass transition temperature. The decrease in the exothermic effect during curing is proportional to the theoretically calculated content of oxazine rings (based on a 100% yield of BA-a monomer and hexasubstituted phosphazene with six oxazine rings). The reduced content of oxazine rings in the BP-3 sample is associated with the presence of a significant amount of oligophosphazenes with a bisphenol bridge in their structure.

Based on the TGA data (Table 6, detailed description is presented in the supplementary information) obtained in the air atmosphere, it can be concluded that the phosphazene component has a small positive effect both on the initial destruction temperatures and on the char yield. At 550–600 °C, the inhibition of theormoxidative degradation caused by the presence of phosphazene is noticeable (Figure S6a). In an inert atmosphere, the behavior of all samples in the range of 300–800 °C is very similar (Figure S6b) but, unusually, with an increase in the amount of phosphazene component (phosphorus) in the composition, a decrease in char yield is observed. This may be caused by the loosening of the polymer network in the presence of more phosphazene. The highest char yield of 37% in an inert medium has the composition BP-1 with a 25% content of the phosphazene component.

The values of the limiting oxygen index (LOI) calculated using the empirical Van-Crevelen-Hovtyzer Equation (1) [39] are 31–32 (Table 5), and are almost independent of the phosphazene content.

LOI = 17.5 + 0.4 CY,(1)

where CY is char yield.

At the maximum phosphazene content, the experimental and calculated LOI values (32) are equal (Table 5). Unusually, both the experimental and calculated values of the LOI are quite low. The reason is probably both the abnormally low char yield values of the phosphazene-containing polybenzoxazines obtained in this work (the latter for all phosphazene-containing polybenzoxazines described in the literature are higher than those obtained in this work [14,20,22,23,24,40]), as well as a low phosphorus content (maximum 2.5%)

Despite this, when the content of phosphazene is more than 30% (1.5% phosphorus) in the compositions (BP-2, BP-3), the material has maximum flammability resistance (V-0 rating) according UL-94. Burning stop times of the samples are listed in Table S1. When burning, there is a slight intumescence, which is more noticeable with an increase in the phosphazene content.

Phosphazene-containing benzoxazines in comparison with poly-BA-a are characterized by increased tensile strength (Table 5). The BP-2-based sample has the highest strength, i.e., 84 MPa (40% more than the unmodified sample). With an even higher phosphazene content (BP-3 sample), the strength decreases, remaining higher than that of the unmodified system. Thus, as in epoxy systems [18] modified with functional phosphazene, a dependence with extremum of the mechanical properties on the content of the modifier is observed, with optimal values for a relatively small phosphazene content. However, in the polybenzoxazine system, to achieve the highest strength, about 30% of the phosphazene modifier is required, while in the epoxy system, only about 10% is required [18].

## 4. Conclusions

The phosphazene-containing functional bisphenol A based benzoxazines obtained in the present work have a phosphazene component content of 20–50% and a phosphorus content of up to 2.5%. These phosphazene-containing benzoxazines polymerize to form polybenzoxazines with improved flame retardance and mechanical properties, while other characteristics are no worse than conventional polybenzoxazines. Thus, the obtained phosphazene-containing benzoxazines may be used as a component of a binder for composite materials.

## Figures and Tables

**Table 1:** The amount of starting reagents for the synthesis of hydroxyaryloxycyclotriphosphazene with a different ratio of HCP: bisphenol A.

Parameter	Unit	Molar Ratio of HCP: Bisphenol A		
		1:24	1:16	1:12
The content of the phosphazene component in a mixture with bisphenol A ^1^	wt%	27	40	52
Designation of benzoxazines synthesized on these mixtures	-	BP-1	BP-2	BP-3
Bisphenol A	g	62.97	42.0	31.5
	mole	0.276	0.184	0.138
Aniline	g	2.78	2.57	2.35
	mole	0.0298	0.0276	0.252
Paraformaldehyde	g	2.068	1.840	1.746
	mole	0.063	0.061	0.058

^1^ Calculated under the assumption that the product consists only of unreacted bisphenol A and a hexasubstituted reaction product of HCF and bisphenol A (P_3_N_3_(OC_6_H_4_C(CH_3_)_2_C_6_H_4_OH)_6_).

**Table 2:** Results of ^1^H NMR spectroscopy of BA-a and phosphazene-containing benzoxazines.

Sample	Phosphazene Content, %	Relative ^1^H Signal Intensity			
		Benzoxazine Ring		-CH_2_-N- (Oligomer) ^1^	-CH_3_ of Bisphenol A
		-CH_2_-O-	-CH_2_-N-		
		δ_H_ = 5.37 (s)	δ_H_ =4.62 (s)	δ_H_ = 4.48 (s)	δ_H_ = 1.63 (s)
**BA-a**	0	1.00	1.00	0.02	1.49
**BP-1**	27	1.00	1.01	0.13	2.05
**BP-2**	40	1.01	0.99	0.17	2.39
**BP-3**	52	1.00	1.01	0.29	3.19

^1^ Examples of such oligomers and corresponding aminomethylene Mannich bridges are shown on Figure 2.

**Table 3:** Molecular weight characteristics and composition of phosphazene-containing benzoxazines.

Sample	M_n_	M_w_	M_w_/M_n_	Content (wt%)	
				Phosphorus ^1^	Phosphazene Containing Fraction ^2^
**BA-a**	480	610	1.27	-	-
**BP-1**	570	1240	2.20	0.88/1.09	20.9/25.8/19.9
**BP-2**	570	1830	3.23	1.36/1.61	32.3/38.1/24.7
**BP-3**	910	5800	6.38	1.87/2.47	44.3/58.4/49.8

^1^ Calculated/Found, ^2^ Calculated/Found by elemental analysis/GPC data.

**Table 4:** DSC results of benzoxazines in the curing process.

Sample	Exotherm Characteristics				Relative Benzoxazine Cycles Content ^1^	
	Temperature °C			Polymerization Heat (J/g)		
	Beginning	Maximal Value	End		Calculated	Found ^2^
**BP-3**	181	215	248	247	75.0	71.0
**BP-2**	209	227	250	283	82.0	82.0
**BP-1**	223	234	247	297	87.5	86.0
**BA-a**	233	242	252	346	100.0	100.0

^1^ Regarding the maximum possible, for which the value corresponding to the pure monomer BA-a was taken. ^2^ Found by dividing of polymerization heat value of the sample by polymerization heat of BA-a.

**Table 5:** Thermal and mechanical properties and flammability of polybenzoxazines.

Parameter	Sample			
	BA-a	BP-1	BP-2	BP-3
**Glass transition temperature (°C) ^1^**	168/137	173/132	168/145	169/136
**LOI ^2^**	31/23	32/29	32/31	32/32
**UL-94 Rating**	V-1	V-1	V-0	V-0
**Tensile strength (MPa)**	60	65	84	75

^1^ Preset curing mode/second scan in a DSC, ^2^ Calculated by Van Krevelen and Hoftyzer equation/Determined by ASTM D2863.

**Table 6:** The results of thermogravimetric analysis of polymers based on BA-a and phosphazene-containing benzoxazines.

Parameter	In Air				In Argon			
	BA-a	BP-1	BP-2	BP-3	BA-a	BP-1	BP-2	BP-3
**Temperature of weight loss (°C)**								
**Beginning**	290	302	312	315	285	303	320	303
**T_5%_**	318	330	338	336	314	324	338	336
**T_10%_**	351	365	366	363	347	349	363	359
**Char yield (wt%)**								
**at 600 °C**	22	32	35	35	35	40	39	38
**at 700 °C**	0	9	18	19	34	38	38	36
**at 800 °C**	0	1	2	2	33	37	36	35

## Supplementary Materials

The following are available online at https://www.mdpi.com/2073-4360/12/6/1225/s1, Figure S1. ^31^P NMR spectrum of phosphazene-containing benzoxazines at HCF: bisphenol A ratios of 1:24 (spectra at ratios of 1:12, 1:16, 1:24 are identical). Figure S2. ^1^H NMR spectrum of the phosphazene-containing benzoxazine obtained at a ratio of HCF: bisphenol A = 1:24. Figure S3. 1H NMR spectrum of the phosphazene-containing benzoxazine obtained at a ratio of HCF: bisphenol A = 1:16. Figure S4. 1H NMR spectrum of the phosphazene-containing benzoxazine obtained at a ratio of HCF: bisphenol A = 1:12. Figure S5. ESI + mass spectra of phosphazene-containing benzoxazine. Retention time: A—13.7 min, B—15.1 min, C—16.4 min, D—17.8 min. Figure S6. TGA curves of BA-a and phosphazene-containing benzoxazines based polymers obtained in air (a) and in argon (b). Table S1. Determining the flammability class of samples according to UL-94 standard. Figures S7–10—^1^H NMR spectra of BA-a and phosphazene-containing benzoxazine obtained at a ratios of HCF: bisphenol A of 1:24, 1:16 and 1:12 respectively.
