# Supplementary material for: Synthesis of Phosphazene-Containing, Bisphenol A-Based Benzoxazines and Properties of Corresponding Polybenzoxazines
Source: Polymers (Basel). 2020 May 28;12(6):1225. doi: 10.3390/polym12061225 (PMC7361955; doi:10.3390/polym12061225)
Supplement: Supplementary file 1 [file polymers-12-01225-s001.pdf]

polymers

Supplementary information

Synthesis of Phosphazene-Containing, Bisphenol A-Based Benzoxazines and Properties of Corresponding Polybenzoxazines

Igor S. Sirotin *, Igor A. Sarychev, Viktoria V. Vorobyeva, Anastasia A. Kuzmich, Natalia V. Bornosuz, Denis V. Onuchin, Irina Yu. Gorbunova and Vyacheslav V. Kireev

Mendeleev University of Chemical Technology of Russia, 125047, Miusskaya sq. 9, Moscow 125047, Russia; yahoo123-92@mail.ru (I.A.S.); vorobyevavv1995@mail.ru (V.V.V.); akuzmich@muctr.ru (A.A.K.); bornosuz@muctr.ru (N.V.B.); donuchin@muctr.ru (D.V.O.); igorbunova@muctr.ru (I.Y.G.); kireev@muctr.ru (V.V.K.)

* Correspondence: isirotin@muctr.ru; Tel.: +7-(499)-978-91-98

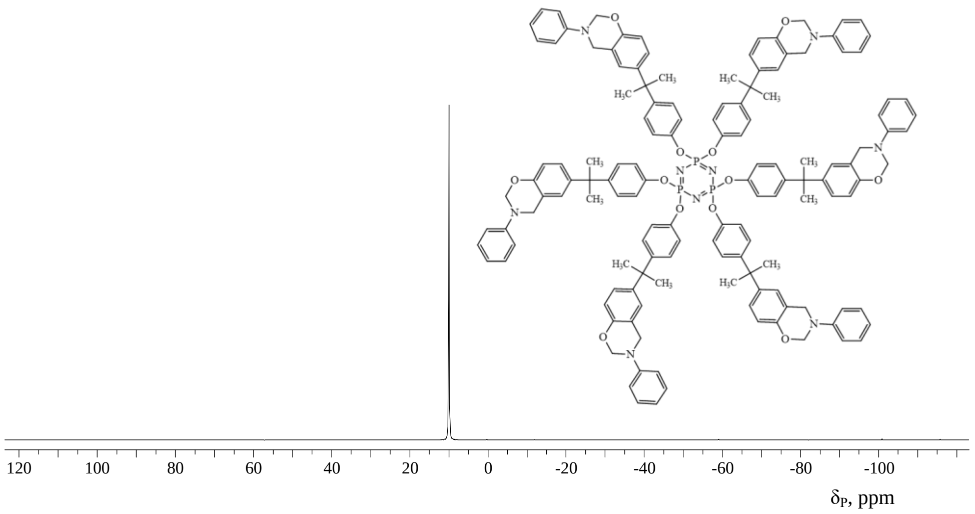

Figure S1. ^{31}P NMR spectrum of phosphazene-containing benzoxazines at HCP:bisphenol A ratio of 1:24 (spectra at ratios of 1:12, 1:16, 1:24 are identical).

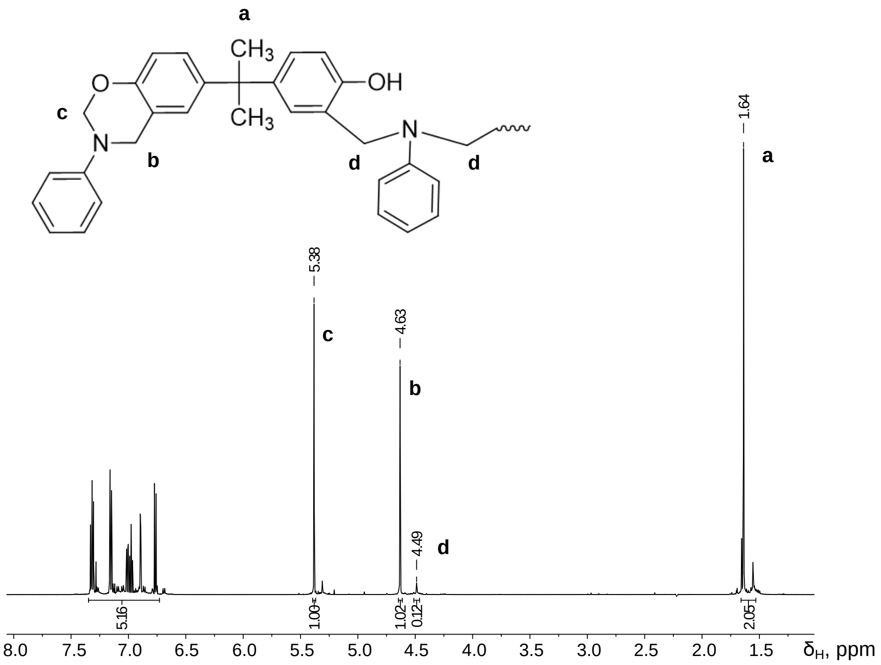

Figure S2. ^{1}H NMR spectrum of the phosphazene-containing benzoxazine obtained at a ratio of HCF: bisphenol A = 1:24.

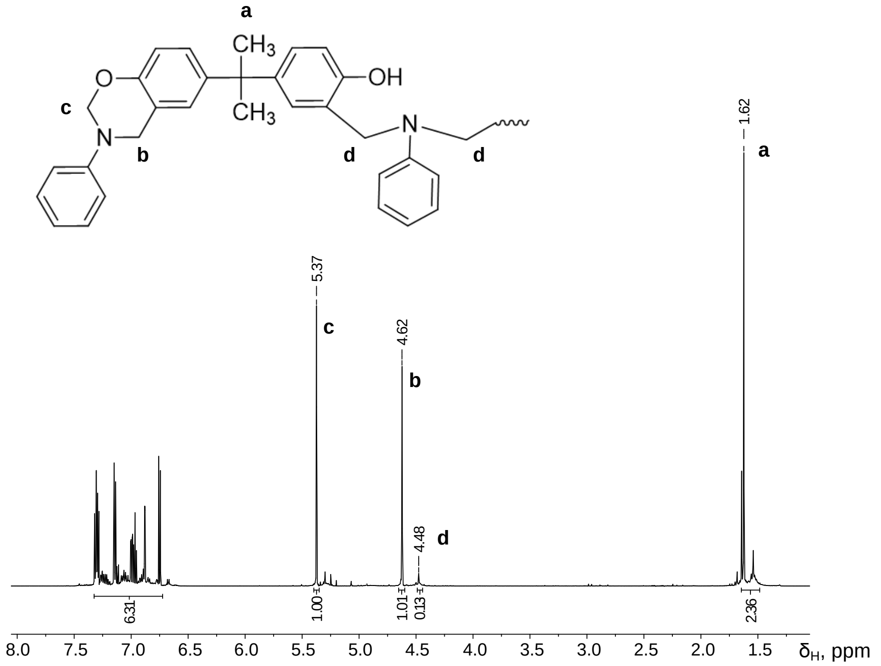

Figure S3. ^{1}H NMR spectrum of the phosphazene-containing benzoxazine obtained at a ratio of HCF: bisphenol A = 1:16.

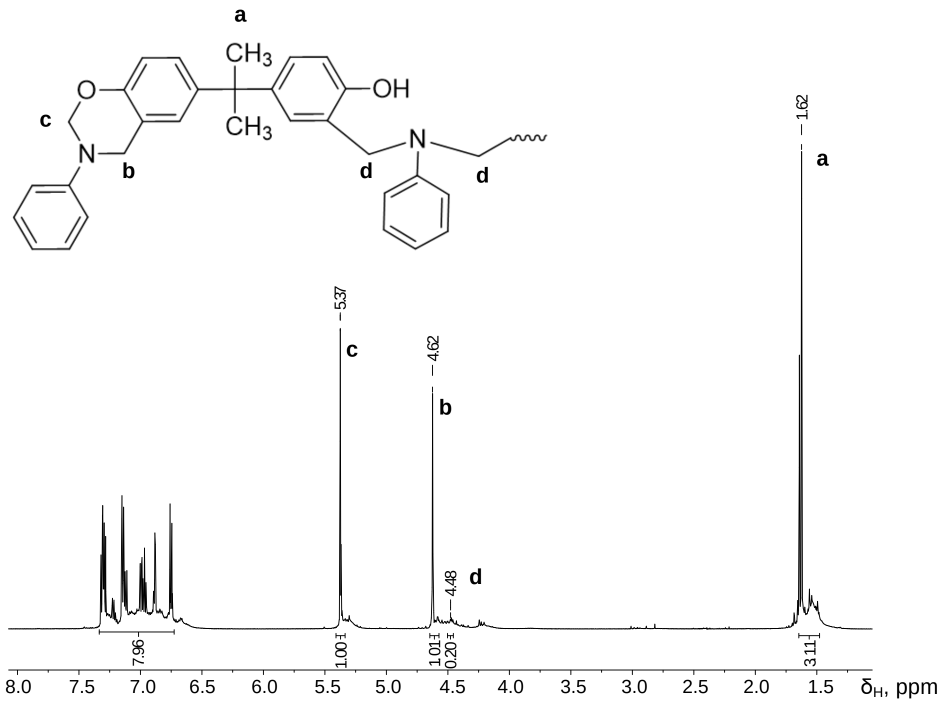

Figure S4. ^{1}H NMR spectrum of the phosphazene-containing benzoxazine obtained at a ratio of HCF: bisphenol A = 1:12.

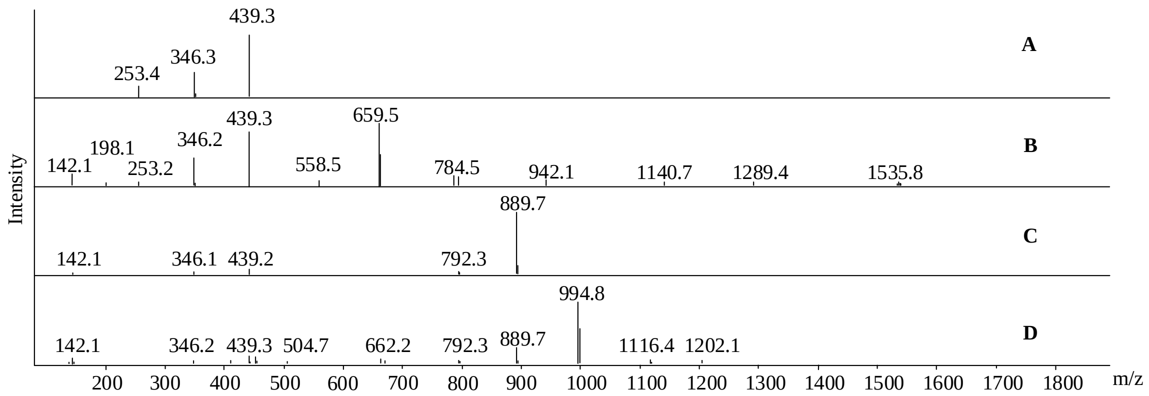

Figure S5. ESI [+] mass spectra of phosphazene-containing benzoxazine. Retention time: A - 13.7 min, B - 15.1 min, C - 16.4 min, D - 17.8 min.

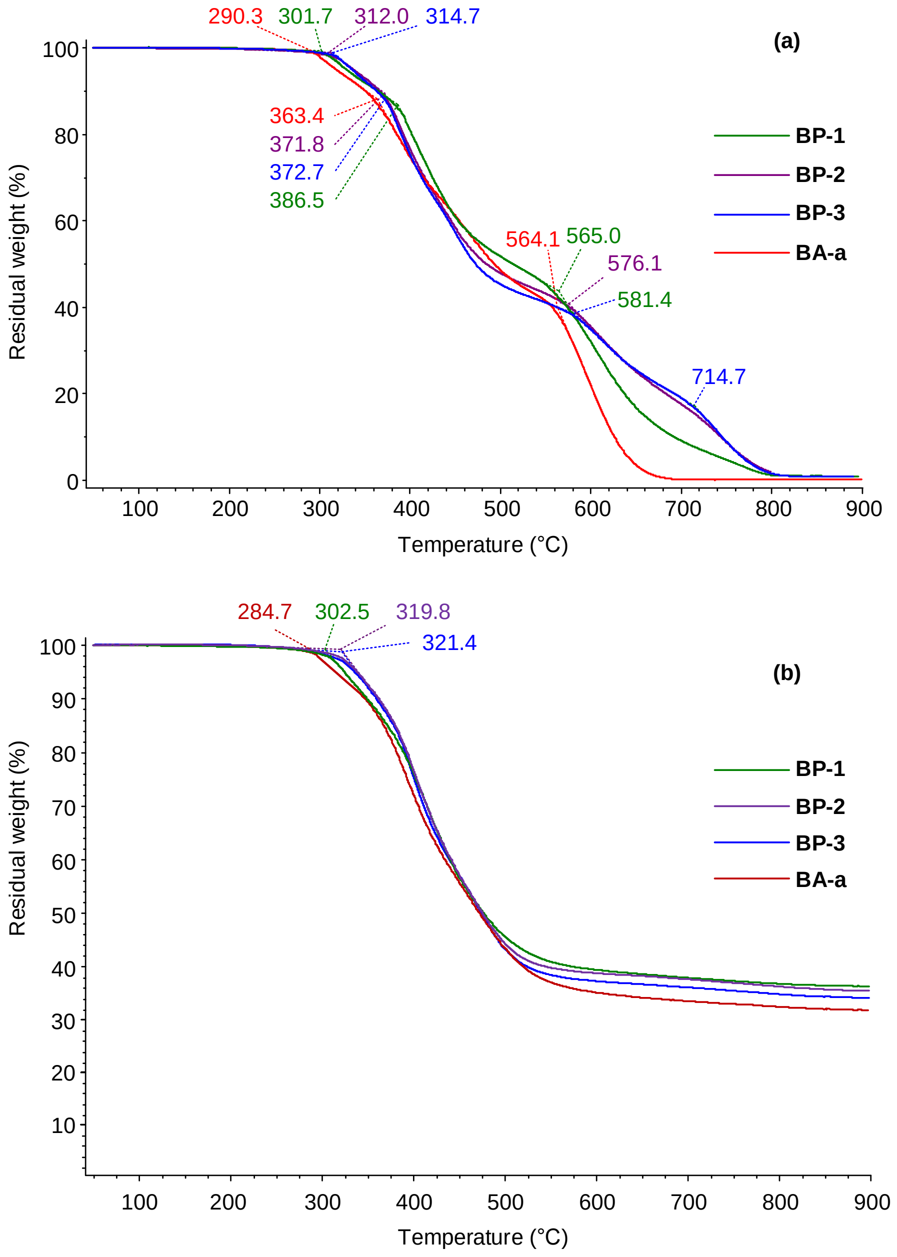

Figure S6. TGA curves of BA-a and phosphazene-containing benzoxazines based polymers obtained in air (a) and in argon (b).

TGA description

Based on the TGA data (Table 6, the paper) obtained in the air atmosphere (Figure S6a), it can be concluded that the phosphazene component has a small positive effect both on the initial destruction temperatures and on the coke residue. The temperatures of 5% weight loss, compared to BA-a monomer, are 11.5, 19.2 and 17.5 °C higher for samples BP-1, BP-2 and BP-3, respectively. The temperatures of the 10% mass loss, with an increase in the content of the phosphazene fraction, practically do not differ and increased only by 12.2–15.5 °C. However, with an increase in the content of the phosphazene component in the compositions, the nature of the curves slightly differs from the BA-a monomer (Figure 6, Supplementary Information). On the TGA curves there are several characteristic temperature ranges, corresponding, in our opinion:

1) 310-380 °C corresponds to the destruction of methyl groups in bisphenol A;

2) 350-500 °C presumably corresponds to the destruction of both methylene bridges, methyl groups in bisphenol A and, probably, benzene rings of aniline. For BA-a, the temperature of the beginning of the transition to the second stage of destruction is 362 °C, and for samples BP-1, BP-2,

and BP-3 - 395, 372, and 376 °C, respectively. The introduction of phosphazene cycles contributes to an increase in the temperature of intense destruction, however, the magnitude of this effect varies nonlinearly with an increase in the content of the phosphazene fraction. This can be explained by the presence in phosphazene of a different number of cyclolinear oligomers, which negatively affect the rate of destruction. The highest temperature of intense destruction is characteristic of BP-1.

3) 550-600 °C – intense oxidation. For BA-a monomer, the transition to intense oxidation occurs at 558 °C, and for BP-1 samples; BP-2 and BP-3 - 567; 573 and 583 °C, respectively. At this stage, the most noticeable positive effect of phosphazene, which inhibits the thermal oxidative degradation of the polymer. The most resistant to thermal oxidative degradation were BP-3 and BP-2.

Table S1. Determining the flammability class of samples according to UL-94 standard.

**Sample**	**Burning duration after the first flame exposure, s**	**Burning duration after the second flame exposure, s**	**Drop formation**	**Flammability class**
**BA-a**	15	3,5	no	V-1
**BP-1**	11	1	no	V-1
**BP-2**	3	7	no	V-0
**BP-3**	7	10	no	V-0

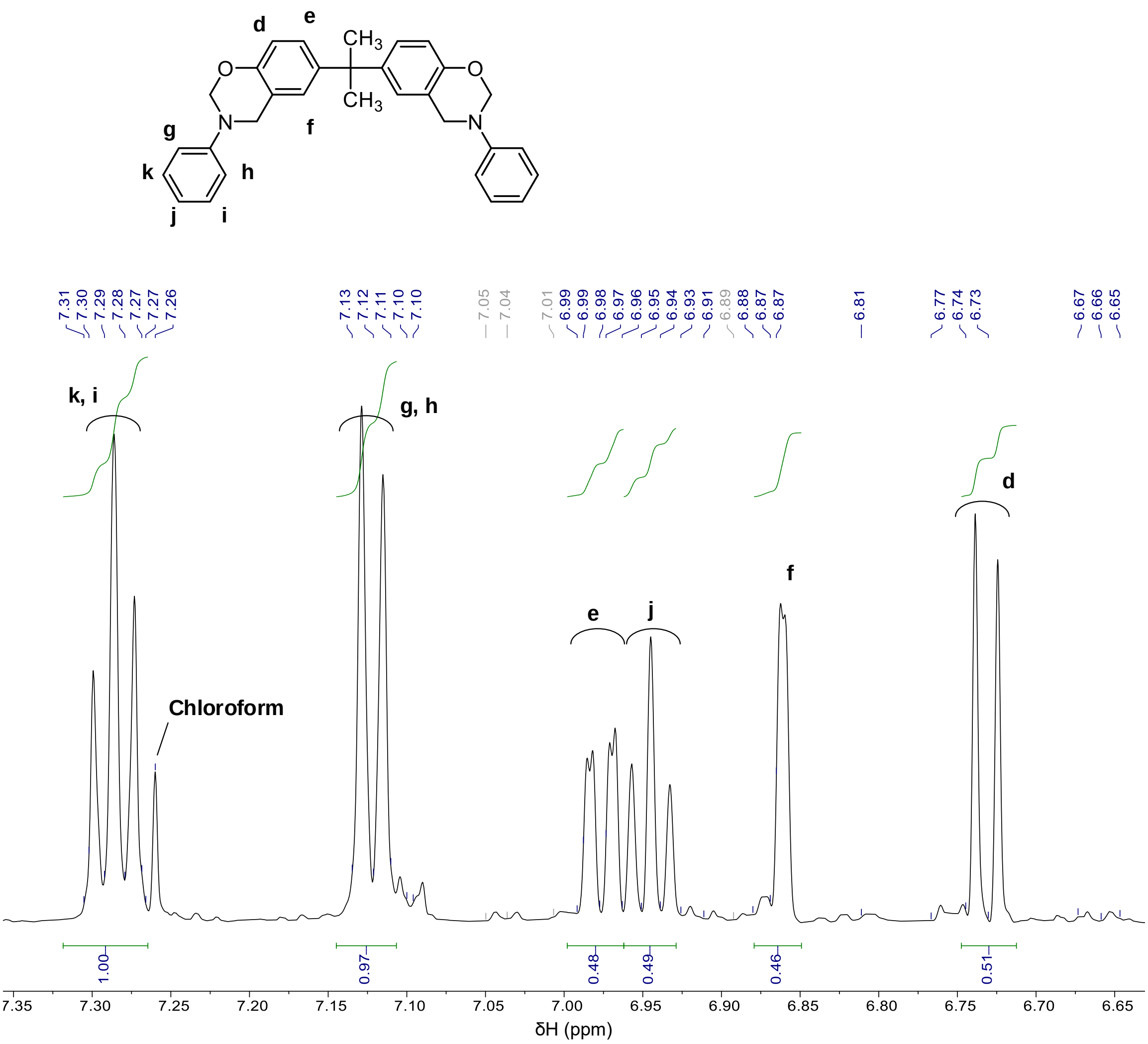

Figure S7. ^{1}H NMR spectrum of the BA-a (region corresponding to aromatic moieties).

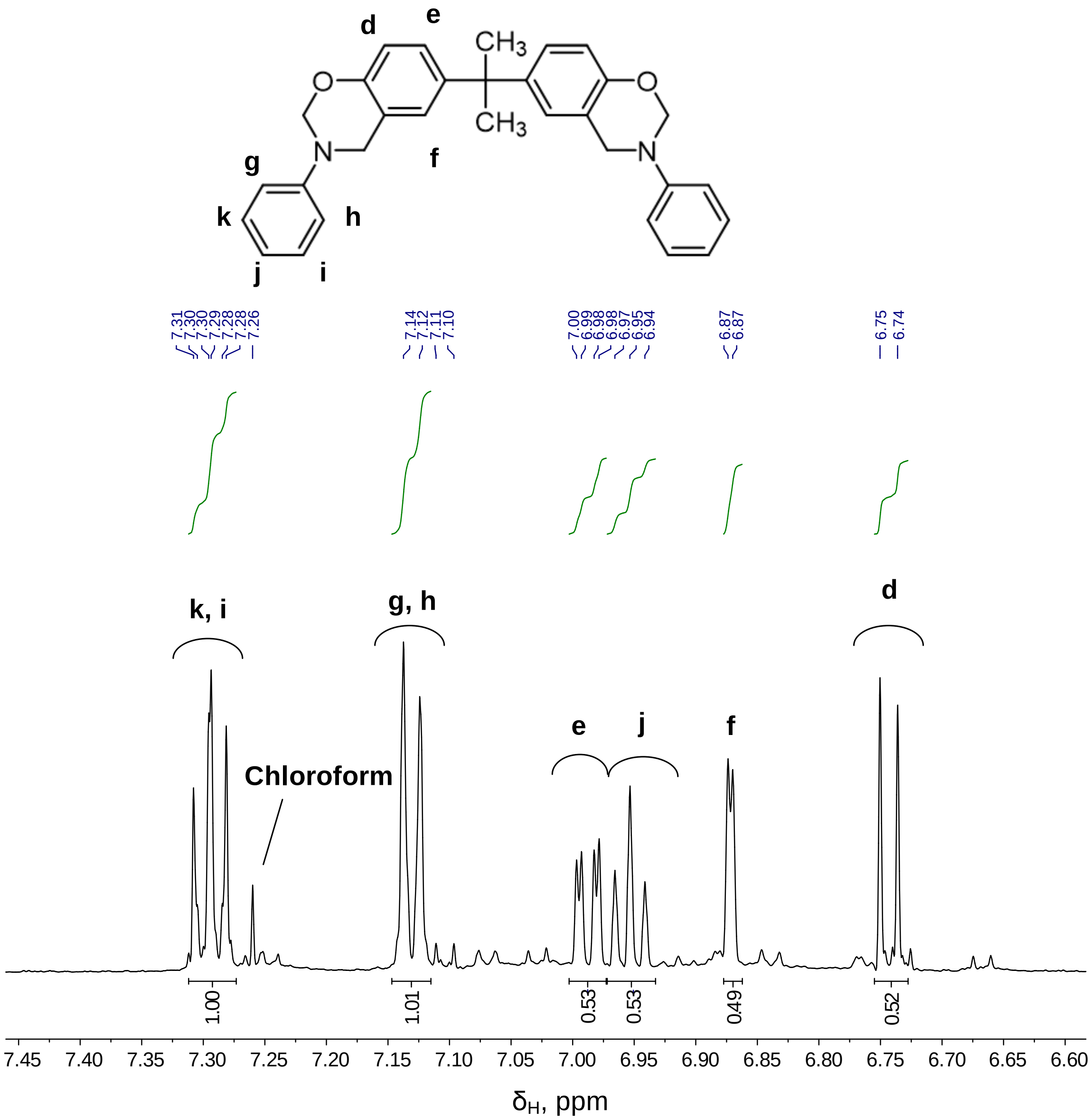

Figure S8. ^{1}H NMR spectrum of the phosphazene-containing benzoxazine obtained at a ratio of HCF: bisphenol A = 1:24 (region corresponding to aromatic moieties).

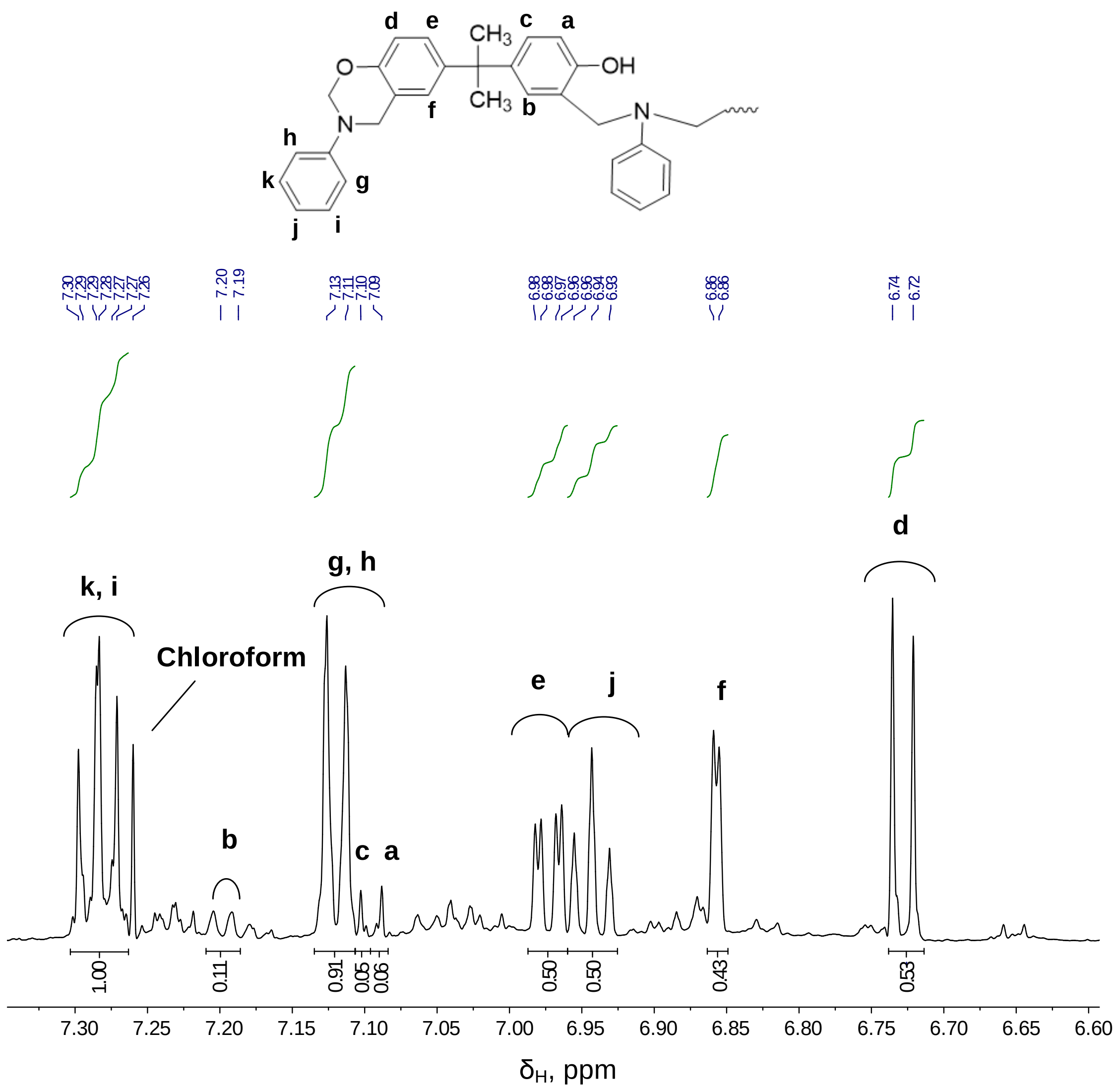

Figure S9. ^{1}H NMR spectrum of the phosphazene-containing benzoxazine obtained at a ratio of HCF: bisphenol A = 1:16 (region corresponding to aromatic moieties).

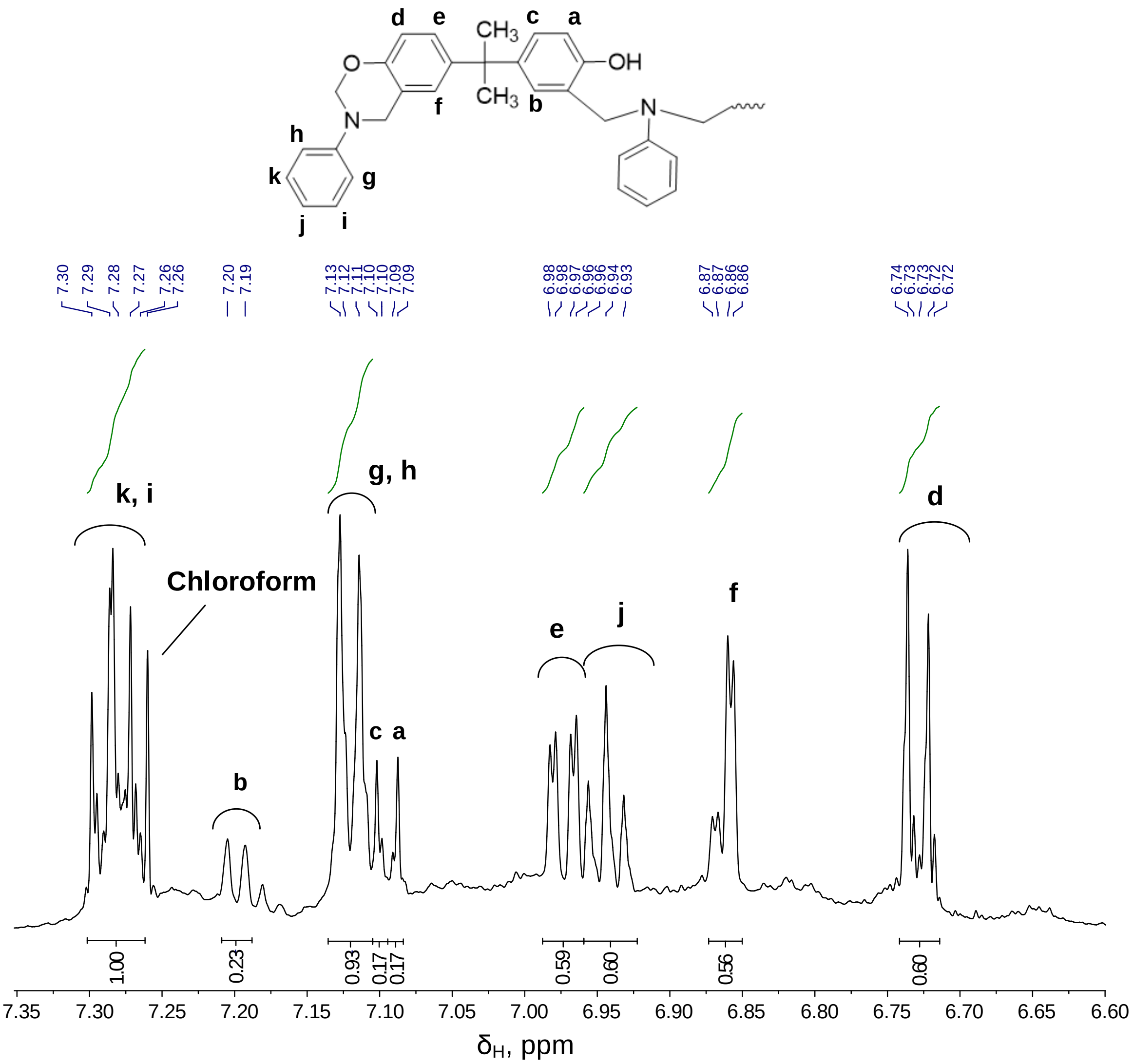

Figure S10. ^{1}H NMR spectrum of the phosphazene-containing benzoxazine obtained at a ratio of HCF: bisphenol A = 1:12 (region corresponding to aromatic moieties).

© 2020 by the authors. Submitted for possible open access publication under the terms and conditions of the Creative Commons Attribution (CC BY) license (http://creativecommons.org/licenses/by/4.0/).
